# A Case of Fulminant Fusobacterium necrophorum Bacteremia Secondary to Non-severe COVID-19

**DOI:** 10.7759/cureus.35717

**Published:** 2023-03-03

**Authors:** Hiroaki Taniguchi, Takero Terayama, Nobuaki Kiriu, Susumu Matsukuma, Tetsuro Kiyozumi

**Affiliations:** 1 Traumatology and Critical Care Medicine, National Defense Medical College, Tokorozawa, JPN; 2 Pathology and Laboratory Medicine, National Defense Medical College, Tokorozawa, JPN

**Keywords:** secondary infection, coronavirus, sars-cov-2, bacterial infection, omicron variant, fusobacterium necrophorum, covid-19

## Abstract

The Omicron variant of severe acute respiratory syndrome coronavirus 2 (SARS-CoV-2) is more infectious than the previous variants but less severe; more patients are being followed up without hospitalization. Identification of patients with severe disease symptoms as early as possible and prompt initiation of treatment are crucial. A case of a 19-year-old man with mild COVID-19 is described in this report. He died of a secondary infection with Fusobacterium necrophorum bacteremia and a progressive hemorrhagic disorder. The diagnosis was made based on the clinical course and needle necropsy results. In non-severe COVID-19 patients, rapid deterioration of the disease symptoms requiring emergency treatment should lead to suspicion of additional fatal infections with similar clinical symptoms.

## Introduction

The clinical symptoms of COVID-19 differ among the variants [1]. While previous variants of severe acute respiratory syndrome coronavirus 2 (SARS-CoV-2), such as the delta variant, are characterized by shortness of breath, runny nose, and fever, the Omicron variant of SARS-CoV-2 is characterized by upper respiratory tract symptoms such as sore throat and a lower risk of critical illness [1]. Patients with mild COVID-19 who do not require supplemental oxygen therapy are generally treated at home. Some risk factors for severe disease that have been reported so far include older age, diabetes mellitus, and an immunocompromised host.

We report an unusual case of a mildly ill COVID-19 patient, without a high risk of serious illness, who died soon after admission due to a Fusobacterium necrophorum infection. This experience suggests that even for patients with mild COVID-19 without risk factors, there is a need to remain alert for additional serious bacterial infections, and prompt intervention should be initiated before their condition worsens.

## Case presentation

A 19-year-old man was diagnosed with COVID-19 by a reverse transcription-polymerase chain reaction (RT-PCR) test for SARS-CoV-2, a day after the onset of fever. He had no past medical history of illness and had already been administered two doses of the Pfizer-BioNTech COVID-19 vaccine (BNT162b2). He was followed up at home because of a mild illness with no risk of progressing to severe COVID-19. However, his clinical symptoms gradually deteriorated, with a sore throat, fever (with a body temperature of over 40°C), and chills. Five days after onset, he presented with impaired consciousness and was transferred to a nearby acute-care hospital. He was intubated, and norepinephrine was administered owing to severely impaired consciousness and hypotension. A whole-body computed tomography (CT) scan at the hospital revealed neither any signs of pneumonia nor any other findings as the source of the fever. The patient was transferred to our tertiary care center because intensive care was required.

On arriving at our hospital, a physical examination revealed a consciousness disturbance with a Glasgow Coma Scale (GCS) of E1VTM4. The heart rate was 180 beats per minute, and the blood pressure was 94/69 mmHg under continuous infusion of 0.1 µg/kg/min of norepinephrine. Initial blood examination revealed severe inflammation, acute kidney injury (AKI), and severe coagulopathy. Arterial blood gas analysis revealed metabolic acidosis (Table 1).

**Table 1:** Laboratory findings at admission to the ICU Abbreviations: ICU: intensive care unit; WBC: white blood cell count; RBC: red blood cell count; Hb: hemoglobin; Hct: hemocytocrit; Plt: platelet count; T-Bil: total bilirubin; AST: aspartate aminotransferase; ALT: alanine aminotransferase; LDH: lactate dehydrogenase; ALP: alkaline phosphatase; TP: total protein; ALB: albumin; BUN: blood urea nitrogen; CRP: C-reactive protein; APTT: activated partial thromboplastin time; PT: prothrombin time; ADAMTS-13: disintegrin-like metalloprotease with thrombospondin type 1 motif 13 * Blood gas analysis was performed under the following conditions of invasive mechanical ventilation: pressure-controlled assist/control (AC/PC) mode (FiO2, 0.5; positive end-expiratory pressure [PEEP], 5 cmH2O; inspiratory pressure above PEEP [PC], 12 cmH2O; ventilator respiratory rate, 15 breaths/min)

Variables	Results	Normal range
Arterial blood gas analysis*		
pH	7.296	7.380 ~ 7.460
PaO_2_ (mmHg)	117	74.0 ~ 108.0
PaCO_2_ (mmHg)	38.5	32.0 ~ 46.0
HCO_3_ (mmol/L)	18.2	21.0 ~ 29.0
Base excess (mmol/L)	-7.3	-2.0 ~ 2.0
Lactate (mmol/L)	7.4	0.4 ~ 1.6
Complete blood cell count		
White blood cell count (×10^3^/μL)	8.0	3.3 ~ 8.6
Red blood cell count (×10^6^/μL)	4.07	4.35 ~ 5.55
Hemoglobin (g/dL)	12.8	13.7 ~ 16.8
Hematocrit (%)	37.2	40.0 ~ 50.0
Platelet count (×10^4^/μL)	0.9	15.8 ~ 34.8
Blood biochemistry		
Total bilirubin (mg/dl)	0.92	0.2 ~ 1.2
AST (U/L)	702	8 ~ 30
ALT (U/L)	262	5 ~ 35
LD (U/L)	2170	100 ~ 225
ALP (U/L)	127	38 ~ 113
Total protein (g/dL)	5.5	6.5 ~ 8.2
Albumin (g/dL)	2.6	3.8 ~ 5.2
BUN (mg/dL)	29	8 ~ 20
Creatine (mg/dL)	2.42	0.61 ~ 1.13
Sodium (mmol/L)	140	135 ~ 147
Potassium (mmol/L)	2.9	3.5 ~ 5.0
Chlorine (mmol/L)	102	98 ~ 108
Procalcitonin (ng/mL)	> 100	< 0.05
C-reactive protein (mg/dL)	13.3	< 0.3
Blood coagulation		
Prothrombin time activity assay (%)	below sensitivity	80 ~ 127
Activated partial thromboplastin time (sec)	133.7	24.0 ~ 32.0
Fibrinogen (mg/dL)	< 30	180 ~ 400
Immunological tests		
Heparin-induced thrombocytopenia antibody (U/mL)	< 0.6	< 1.0
Direct coombs test	negative	negative
ADAMTS13	negative	negative
C_3 _(mg/dL)	99	65 ~ 135
C_4 _(mg/dL)	39	13 ~ 35

The patient was initially diagnosed with severe COVID-19 and disseminated intravascular coagulation (DIC). The sequential organ failure assessment score was 13 (respiration: one, coagulation: four, liver: 0, cardiovascular: three, central nervous system: three, renal: two). The DIC score established by the Japanese Association for Acute Medicine [2]-the criteria established for the diagnosis of DIC-was nine points. The patient presented with refractory nasal hemorrhage due to severe coagulopathy, and hemostasis was finally achieved 12 hours after admission with cauterization by an otolaryngologist and a massive blood transfusion. Cefazolin was administered after these procedures.

**Figure 1:**
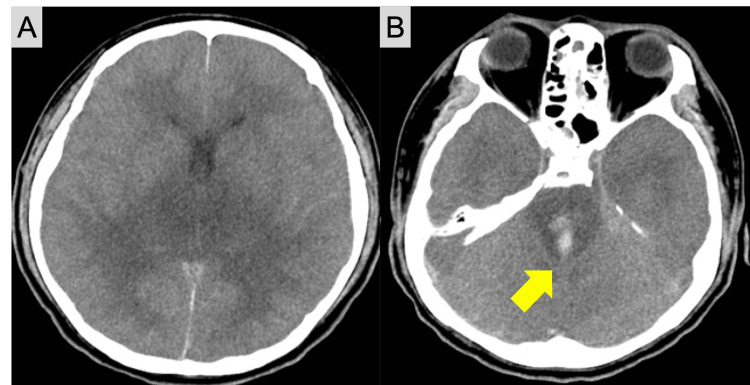
Head computed tomography (CT) scan after the resuscitation The findings of the head CT scan after the resuscitation demonstrated diffuse cerebral edema (A) and intracranial hemorrhage (arrow) in the brain stem, which expanded to the fourth ventricle (B).

After these treatments, an additional CT scan of the head was performed, which revealed diffuse cerebral edema and intracranial hemorrhage in the brainstem extending to the fourth ventricle (Figure 1).

The patient died of increased intracranial pressure due to a brain hemorrhage, two days after admission. The two initial sets of blood bacterial cultures were found to be positive for F. necrophorum.

**Figure 2:**
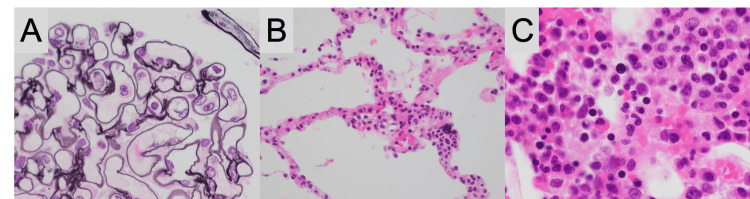
Histopathological images of needle necropsies of the kidney (A), lung (B), and bone marrow (C) (A) A few neutrophils within renal glomerular capillaries with no convincing feature of thrombotic angiopathy, such as thromboendothelial swelling (periodic acid-methenamine silver stain, ×1,000); (B) thickened pulmonary alveolar septa with neutrophils, suggesting septic pneumonitis (hematoxylin-eosin stain, ×400); (C) hypercellular bone marrow without a feature of hemophagocytic syndrome (hematoxylin-eosin stain, ×1,000)

Post-mortem needle biopsies of the kidney, lung, and bone marrow revealed glomerular neutrophils, interstitial neutrophils in the alveolar septum, and hypercellular bone marrow, respectively (Figure 2).

There were no convincing features of thrombotic angiopathy or hemophagocytic syndrome. Considering the clinical course and the needle necropsy results, the patient was finally diagnosed with DIC and septic shock due to F. necrophorum bacteremia secondary to COVID-19.

## Discussion

This case provides us with an important message: when non-severe COVID-19 patients without any risk factors present with rapid deterioration of the disease symptoms requiring emergency treatment, suspicion must be raised for additional fatal infections that are associated with COVID-19 infection, exemplified by mucormycosis and pulmonary aspergillosis [3,4].

According to a survey by the National Institute of Infectious Diseases in Japan, 96.6% of COVID-19 cases in Japan during February 2022 were caused by the Omicron variant; the present case occurred during this period [5]. The Omicron variant is highly infectious but less symptomatic [1]. In Japan, non-severe COVID-19 patients with SpO2 >96% are generally not recommended for hospitalization but rather are advised to receive home care to reduce the occupancy of hospital beds, which are needed for more critical patients. The clinical characteristics of the Omicron variant require an appropriate support system to identify patients with non-severe COVID-19 developing severe conditions and a manifest direction to hospitalize severe patients without delay.

Fever and sore throat are closely associated with upper airway infection caused by flu viruses, Group A Streptococcus, and F. necrophorum [6]. Among them, F. necrophorum infection is more common in healthy young adults, especially males, and is endemic to the mid-pharynx, the gastrointestinal tract, or the vagina; some cases are asymptomatic, but fever is the most common clinical manifestation (39%), followed by a sore throat (13.6%) [7]. Severe complications have also been reported in 38% of Fusobacterium bacteremia: thrombosis (18%), abscess (16%), and Lemierre syndrome (10%) [7]. Lemierre syndrome has been in focus again in recent times as a fatal disease because of the trend of avoiding prescribing antibiotics for mild laryngitis [8]. The present reported mortality rates are 5%-11% [7,9,10]. In this case, too, there was a high suspicion of Lemierre syndrome; however, the diagnosis was not made.

The diagnosis of F. necrophorum was delayed till the end because the symptoms of SARS-CoV-2 (Omicron variant) infection are almost the same as those for F. necrophorum. A throat swab was not sent for culture sensitivity. COVID-19 medication was not administered; only antipyretics were administered. Therefore, the administration of appropriate antibiotics was delayed. Careful diagnosis is necessary so as not to miss lethal bacterial infections that overlap with COVID-19 symptoms, as was the case in this report.

## Conclusions

Here, we report a fatal case of severe F. necrophorum infection secondary to mild COVID-19. Our case emphasizes the importance of diagnosing overlapping severe bacterial infections with similar clinical symptoms as COVID-19 during the treatment of patients with COVID-19, even though the patient may not have any risk factors. This would help in the initiation of prompt intervention before the patient’s condition deteriorates.
